# A 10-Year Study on Percutaneous Cholecystostomy for Acute Cholecystitis at a Tertiary Referral Hospital

**DOI:** 10.3390/jcm15020413

**Published:** 2026-01-06

**Authors:** Margarita Ptasnuka, Ita Lazdane, Vladimirs Fokins, Oksana Kolesova, Haralds Plaudis

**Affiliations:** 1 Department of General and Emergency Surgery, Riga East Clinical University Hospital, LV-1038 Riga, Latvia; ita.lazdane@gmail.com (I.L.); vladimirsfokins@gmail.com (V.F.); hplaudis@gmail.com (H.P.); 2 Department of Surgery, Rīga Stradiņš University, LV-1007 Riga, Latvia; 3 Department of Doctoral Studies, Rīga Stradiņš University, LV-1007 Riga, Latvia; 4 Institute of Microbiology and Virology, Department of Infectology, Rīga Stradiņš University, LV-1069 Riga, Latvia; oksana.kolesova@rsu.lv

**Keywords:** acute cholecystitis, cholecystostomy, cholecystitis in older adults

## Abstract

**Background**: Percutaneous cholecystostomy (PC) is an effective, minimally invasive treatment for patients with acute cholecystitis (AC) who are at high surgical risk and may be used as a bridge to surgery in critically ill patients. This study aimed to evaluate the safety of PC in patients with AC over a 10-year period. **Methods**: Patients who underwent PC for AC at our institution between January 2013 and May 2023 were included. Patients were categorised into the definitive and bridging PC groups. Clinical characteristics, procedure-related complications, recurrence, and overall survival were analysed. Statistical analyses were used to identify in-hospital mortality-related risk factors. **Results**: A total of 449 patients were included, and 89.5% had an ASA score ≥ 3. The median time to PC was 1 day, and 17.6% of patients required ICU admission. Drainage tube-related complications occurred in 37 (8.2%) patients. The median drainage and hospital stay durations were 9 (IQR 6–14) and 12 (IQR 9–15) days, respectively. During follow-up, recurrent AC was observed in 34 (7.6%), with a median time to recurrence of 63 (IQR 29–312) days. PC was the definitive treatment in 275 (61.2%) patients. The overall mortality rate was 5.3% (n = 24), with no deaths related to the drainage procedure. Sepsis on admission was an independent risk factor related to in-hospital mortality. **Conclusions**: Our findings confirmed that PC is a safe and effective treatment alternative for managing AC in high-risk patients with low complication and mortality rates.

## 1. Introduction

Acute cholecystitis (AC), a common complication of symptomatic gallstone disease, remains a leading cause of acute surgical admissions worldwide, accounting for approximately 3–10% of all hospitalisations due to acute abdominal pain [1]. With the aging global population and increasing prevalence of gallstone disease among older patients, the incidence of AC is expected to impose an increasing burden on healthcare systems in the following decades [2,3].

According to the Tokyo Guidelines 2018 (TG18), early laparoscopic cholecystectomy (LC) performed within 72 h of symptom onset is the gold standard for AC treatment [4]. Early LC is associated with the lower rates of complication, length of hospital stay, and mortality [5,6]. However, the management of AC in frail or critically ill patients remains challenging, as surgical intervention—even when performed using a minimally invasive approach—is associated with increased morbidity and mortality.

Percutaneous cholecystostomy (PC) is an effective, minimally invasive option for high-risk patients with AC who are not candidates for immediate surgery [7,8]. PC enables gallbladder decompression, rapid source control, sepsis management, and stabilisation of the patient’s condition. It may serve as a bridging procedure before delayed or interval cholecystectomy or as a definitive treatment for patients who cannot undergo surgery. In clinical practice, the rate of cholecystectomy after PC varies widely, ranging from 7.2% to 66.7%, reflecting differences in institutional protocols and patient selection [9,10]. Despite the years of clinical use, the role of PC remains debatable. Notably, international consensus remains limited; the E-AHPBA, ANS, and WSES Delphi consensus achieved agreement on only six key statements regarding PC management [11]. According to a recent systematic review and meta-analysis by Cirocchi et al., patients who underwent cholecystectomy had better long-term outcomes, whereas those managed with PC as definitive treatment had higher rates of readmission and recurrent AC [12].

Reported outcomes reveal heterogeneity regarding timing of drain placement and follow-up protocols after PC across populations and healthcare systems. Data from the Baltic region are limited, and regional studies may provide valuable insights into treatment strategies, resource allocation, and patient outcomes.

The primary aim of this study was to evaluate the safety of PC in patients with AC at a tertiary referral hospital over a 10-year period. The secondary aims were (1) to analyse the outcomes of surgical treatment in patients who subsequently underwent cholecystectomy after PC and (2) to identify factors associated with long-term survival. This study sought to provide new evidence on the role of PC in the management of AC and offer insights into regional clinical practices and treatment outcomes in high-risk patient populations.

## 2. Materials and Methods

### 2.1. Study Design and Patient Population

**Figure 1:**
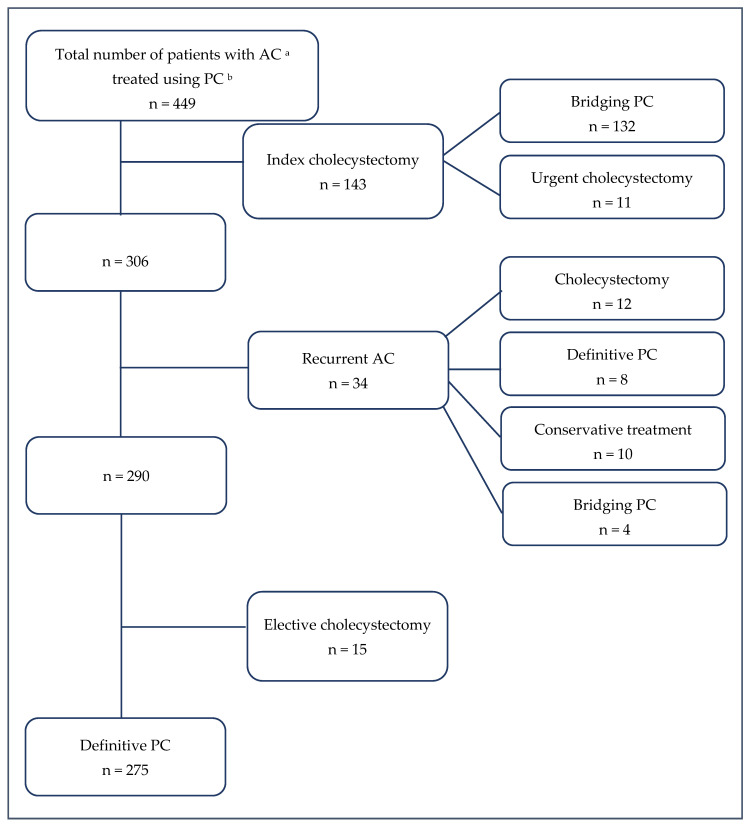
Flow diagram of patients treated with PC. ^a^ AC: Acute cholecystitis. ^b^ PC: Percutaneous cholecystostomy.

All patients who underwent PC for AC at Riga East Clinical University Hospital between January 2013 and May 2023 were included in the study. Patients who received PC for other indications, such as cholangitis or acute pancreatitis, were excluded. AC was defined and diagnosed according to the TG18 criteria [13]. Patients were categorised into two groups based on treatment following PC: (1) the definitive PC group, including patients who did not undergo cholecystectomy after initial PC, and (2) the bridging PC group, comprising patients who underwent cholecystectomy after clinical improvement, either during the same admission or as elective surgery. Figure 1 presents the flow diagram of the study population and outcomes.

### 2.2. PC Technique

All patients were evaluated by the clinical HPB team before PC intervention. Written informed consent was obtained from the participants, whenever possible. For patients who were unconscious or clinically unstable due to severe sepsis, and informed consent could not be obtained, PC was performed as a life-saving procedure based on vital indications, in accordance with the national medical law and ethical standards.

PC was performed under local anaesthesia by surgeons with ultrasonographic expertise. Using sterile technique, a transhepatic route through the seventh or eighth intercostal space was performed under ultrasound guidance. An 8- to 10-Fr pigtail drainage catheter was inserted, and its position within the gallbladder lumen was confirmed using ultrasonography. Infected bile was aspirated for microbiological analysis. In cases of purulent content, saline lavage was performed until relative transparency of the aspirated content was observed. We performed follow-up cholangiography through the drain to assess cystic duct patency and confirmed common bile duct clearance.

### 2.3. Data Collection and Definitions

Patient data, including demographics, laboratory results, and the interval between hospital admission and PC, were collected. The severity of the physical condition of each patient was assessed using the American Society of Anaesthesiologists Physical Status Classification System (ASA). The clinical outcomes included length of hospital stay, rate of intensive care unit (ICU) admissions, duration of drainage, morbidity, and mortality. Procedure-related complications were classified as catheter dislocation, tube occlusion, bile leak, bleeding, and other complications (such as biloma or significant post-procedure pain).

Follow-up continued until August 2024, with documentation of recurrent biliary events and subsequent cholecystectomy. Biliary disease-related events included AC, abscess, common bile duct stones, acute pancreatitis, and pain related to removal of biliary catheter that cannot be managed in an outpatient department. Recurrent AC was defined as another episode of AC occurring between the index PC and interval cholecystectomy. For patients undergoing cholecystectomy, the type of surgery and conversion rate from laparoscopic to open surgery were recorded. Data were extracted retrospectively from patient medical records.

### 2.4. Outcomes

The primary study outcome was the safety of PC, assessed based on procedure-related complications and in-hospital mortality. Secondary outcomes included AC recurrence, readmission due to biliary disease-related events, outcomes of surgical treatment following PC, and overall survival. Long-term outcomes were compared between patients managed with definitive PC and those who underwent interval cholecystectomy. Independent predictors of in-hospital mortality were identified using multivariable Cox regression analysis.

### 2.5. Statistical Analysis

Descriptive statistics were used to summarise demographic and clinical parameters. Continuous variables were expressed as the median value with interquartile range (IQR), and categorical variables were presented as numbers and percentages. Comparisons between patients were made using the Chi-square or Fisher’s exact test for categorical data and the Mann–Whitney U test and t-test for continuous data. Survival analysis was performed using the Kaplan–Meier method and compared using a log-rank test. Cox regression analysis was performed to identify factors independently associated with in-hospital mortality. Multivariate analysis included clinically relevant parameters from univariate analysis with *p* values < 0.1. Hazard ratios (HRs) and 95% confidence intervals (CIs) were presented for all variables. *p* < 0.05 was considered statistically significant.

All statistical analyses were performed using IBM SPSS Statistics version 29.0 for Windows (IBM Corporation, Armonk, NY, USA) and Microsoft Excel (Microsoft Corp., Redmond, WA, USA).

## 3. Results

### 3.1. Patient Characteristics

Between January 2013 and May 2023, a total of 449 patients underwent PC for AC and were included in the study cohort. The median age of the entire cohort was 80 years (IQR 73–85), with a slight predominance of females (56.8%). Most patients scored ASA III (46.1%) or ASA IV (43.4%), indicating a high prevalence of significant comorbidities. At admission, sepsis was present in 23.2% of patients. Regarding disease severity, most patients presented with Grade II AC (62.4%, n = 280), followed by Grade III (22.5%, n = 101), and Grade I (15.1%, n = 68). Laboratory measures at admission showed a median leukocyte count of 13.6 × 10^9^/L (IQR 10.5–18.0) and a median C-reactive protein level of 161.6 mg/L (IQR 60.2–269.7). Acute calculous cholecystitis accounted for 94.4% (n = 424) of patients, whereas acalculous cholecystitis was observed in 5.6% (n = 25). All baseline clinical and laboratory characteristics are summarised in Table 1.

### 3.2. Percutaneous Cholecystostomy: Procedure Characteristics and Short-Term Outcomes

The median time from admission to drain placement was 1 day (IQR 1–2), and early drainage within 48 h was performed in 51.4% of patients. The transhepatic route was used for all PC procedures. Seventy-nine patients (17.6%) required ICU admission for sepsis management and stabilisation of general condition.

The overall complication rate was 8.2% (37/449). Bile leak was the most common procedure-related complication. Eleven patients (2.5%) required emergency cholecystectomy, including 10 patients with bile peritonitis and one patient with haemoperitoneum due to bleeding from the puncture site. Details of procedure-related complications are presented in Table 1.

The median duration of gallbladder drainage during the index admission was 9 days (IQR 6–14), and the median hospital stay was 12 days (IQR 9–15). Sixty-seven patients (14.9%) were discharged with the catheter in place and were recommended to undergo drain removal within 2 weeks at the outpatient department. The remaining patients had the drain removed during subsequent surgery.

The in-hospital mortality rate was 5.3% (24/449). The causes of death were severe sepsis and multiple organ failure in twenty-one patients, pulmonary embolism in two patients, and acute mesenteric ischemia in one patient. None of the deaths were directly related to PC.

### 3.3. Surgical Treatment Following PC

Overall, 174 (38.8%) patients underwent cholecystectomy following PC. Of these, PC as a bridging procedure followed by cholecystectomy during index admission was performed in 132 (75.9%) patients, whereas elective delayed cholecystectomy was performed in 15 (6.6%) patients.

Among the cholecystectomies, 81 patients (46.6%) underwent a laparoscopic approach, 84 (48.3%) had open cholecystectomy, and 9 (5.2%) required conversion surgery. Of note, the bailout procedure was performed in five patients (2.9%) due to severe inflammatory infiltration and adhesions in the Calot’s triangle. Surgical outcomes are summarised in Table 2.

Postoperative complications were observed in six patients (2.9%). Three patients had bile duct injury (BDI), all of which were classified as Strasberg type E. BDIs were identified and repaired with T-tube drain placement intraoperatively. One patient presented with hepatic artery injury and was reoperated on the third postoperative day due to haemorrhagic shock, and one patient with bile leakage from the previous drain site underwent re-laparoscopy.

To evaluate the impact of treatment strategies, we compared outcomes between patients who underwent definitive PC and those who underwent interval cholecystectomy following initial drainage. Patients in the definitive PC group were older and more frequently presented ASA IV (*p* < 0.001). In contrast, in the bridging PC group, the length of hospital stay was longer, and fewer patients required ICU admission; however, these differences were not statistically significant. Notably, the mortality rate was significantly higher in the definitive PC group (8.4% vs. 0.6%, *p* < 0.001). A detailed comparison of baseline characteristics and outcomes between the two groups is presented in Table 3.

### 3.4. Follow-Up: Long-Term Outcomes and Risk Factors for In-Hospital Mortality

After a median follow-up of 67 months (IQR 38.5–86.0), a total of 66 readmissions (14.7%) due to biliary disease-related events were recorded. The causes of readmission are summarised in Table 1. Recurrent AC was observed in 34 patients (7.6%) and treated according to clinical indication (Figure 1). The median time from the initial PC to AC recurrence was 63 days (IQR 29–312). Choledocholithiasis was confirmed by magnetic resonance cholangiopancreatography in seven patients. Of these, five patients underwent endoscopic retrograde cholangiopancreatography (ERCP) with bile duct stone extraction, without subsequent cholecystectomy, as no gallstones were detected in the gallbladder. In the remaining two patients, ERCP with stone extraction was followed by elective cholecystectomy.

The estimated mean survival time was 131.6 months (95% CI, 128.7–134.5). The observed 1-year survival rate was 91.6% for patients managed with PC and 99.4% for those who underwent PC as a bridging procedure followed by cholecystectomy. Patients who underwent definitive PC had significantly lower overall survival rates than those who underwent interval cholecystectomy (*p <* 0.001).

The risk factors associated with in-hospital mortality are shown in Table 4. In univariate Cox regression analysis, the presence of sepsis, a higher AC severity grade, and treatment with PC alone compared to PC followed by surgery had a prognostic effect on in-hospital mortality. Variables with a *p* < 0.1 in univariate analysis were included in multivariate analysis. In multivariable Cox regression analysis, sepsis was an independent risk factor of in-hospital mortality, whereas cholecystectomy following PC was independently associated with a lower risk of death (HR 0.07, 95% CI 0.01–0.51, *p* = 0.009).

## 4. Discussion

This study presents a comprehensive 10-year single-centre review of PC in patients with AC at Riga East Clinical University Hospital and contributes valuable evidence to the limited data from the Baltic region. Our hospital offers healthcare services to a local population of approximately 591,882 individuals and provides a 24 h on-call general surgery service. As the national HPB referral unit, the centre provides consultation and specialised care to patients from across Latvia.

There is an increasing incidence of gallstone disease in Europe, particularly among older adults. Gallstone disease affects approximately 10–15% of adults, and AC develops in up to 40% of these patients [14]. Hospital admission rates for AC and gallstone disease have risen in recent decades, increasing the burden on healthcare systems [3]. These trends highlight the importance of developing effective, personalised treatment strategies for older adults and patients with comorbidities.

In our study, the median patient age was 80 years, and 89.5% were classified as ASA III or IV. This clinical profile of our patients represents limited physiological reserve, consistent with previous studies on patients with frailty [15].

According to TG18 and the World Society of Emergency Surgery guidelines, LC remains the gold standard for AC treatment [3,14]. LC within 72 h of symptom onset is associated with reduced morbidity, shorter hospital stays, and lower costs [5,6]. For high-risk patients who cannot undergo cholecystectomy due to anaesthetic or perioperative risks, PC offers effective source control and sepsis management, with technical success rates >90% and clinical improvement in most cases [16,17]. However, the literature regarding the indications, timing, and follow-up protocols of PC remains highly heterogeneous. At present, there are no accepted guidelines or standardised management algorithms defining the patient selection criteria, optimal use, or postprocedural management of PC in patients with severe AC.

According to the literature, PC is a low-risk, minimally invasive procedure that can serve as a definitive treatment or a bridging procedure, with reported procedure-related complication rates of 10–15% [18]. In our cohort, PC was associated with a low complication rate of 8.2% and no procedure-related mortality. These findings support its use as an appropriate initial treatment strategy for critically ill patients with AC.

The optimal duration of catheter placement following PC remains debatable. Some studies recommend drain placement for at least 6 weeks to allow resolution of inflammation and tract maturation; Noh et al. recommends leaving the PC catheter for 2 weeks after transhepatic approach [17,19]. Moreover, other reports suggest that prolonged drainage exceeding 2 weeks may increase the risk of recurrent AC [20]. The local practice in our hospital is early removal of the catheter once clinical improvement is observed. Cholangiography and drain clamping are routinely performed before removal to confirm cystic duct patency. The median drainage duration in our cohort was 9 days, which is shorter than durations reported in most studies. Despite this, our patients did not demonstrate an increased incidence of bile leak after drain removal, suggested that early drain removal may be safe and effective.

The rate of cholecystectomy following PC in our study was 38.8%, which is consistent with reported ranges of 7.2–66.7% [21,22]. Notably, in our study, patients who underwent cholecystectomy were typically younger and had better physical condition. Of these patients, 75.9% underwent cholecystectomy during the index admission. At our hospital, when clinical improvement allows, cholecystectomy is preferred prior to discharge. This approach is further supported by evidence showing that early cholecystectomy after PC may reduce recurrence and postoperative complications rates [5,6]. Patients discharged with a cholecystostomy drain received outpatient reassessment, including evaluation of anaesthesiologist. Elective cholecystectomy was performed in cases where comorbidities could be stabilised and functional status improved. However, many patients did not achieve sufficient functional recovery to safely undergo delayed elective surgery.

Bile duct injury rates during cholecystectomy for AC are consistently higher than those reported for elective cases, ranging from 0.3% to 2.5% [23,24,25]. In our cohort, the BDI rate was 1.7%, which aligns with previous findings for high-risk cholecystectomies. Notably, all BDIs were identified intraoperatively and managed appropriately. Furthermore, the conversion rate to open surgery was 5.2%, which is lower than the 23% reported in the literature for older patients (aged >70 years) with AC [26]. These findings reflect the importance of individualised treatment strategy and suggest that early cholecystectomy after PC can be safe even in high-risk patients when performed in experienced HPB centres.

Several studies have reported readmission and recurrence rates of 21–37%, with recurrent AC in up to one-third of patients treated with definitive PC [7]. Importantly, recurrence after PC does not always represent procedural failure but rather highlights the heterogeneity of patient populations and management strategies across institutions. In our study, 7.6% of the patients experienced recurrent AC, and 7.1% were readmitted for other biliary events, such as choledocholithiasis, pancreatitis, and liver abscess after cholecystectomy. Our values are notably lower than previously reported. Wang et al. reported that patients with complicated cholecystitis, elevated white blood cell counts, and long PC drainage were more likely to experience recurrence; however, Bhatt et al. found no significant relationship between drainage duration and recurrent AC [27,28]. Most cohort studies report a predominance of acalculous cholecystitis among patients managed definitively with PC, and bridging PC strategies are more common for calculous AC. In contrast, our cohort comprised mainly patients with calculous cholecystitis (94.9%), with a low recurrence rate. These findings suggest that PC can be safely and effectively used as a definitive treatment even in patients with calculous AC when surgical risk is high.

Survival analysis in our study revealed a 5.3% overall mortality rate, with sepsis on admission being a significant predictor of worse outcomes. Conversely, interval cholecystectomy was associated with improved OS. According to Yeo et al., delayed surgery after PC improves survival in some patients [29].

### Limitations

This study has several limitations. First, its retrospective design may introduce selection and information bias. Second, only patients who underwent PC as the initial intervention were included; therefore, the outcomes for those treated with early cholecystectomy were not assessed. Consequently, direct comparative analysis between surgical and PC approaches was not possible. Third, decisions regarding cholecystectomy were made by the attending surgeon without standardised institutional protocols. Despite these limitations, the completeness of data allowed for a reliable evaluation of PC in patients with AC. Prospective studies with standardised treatment pathways are needed to better assess the role and long-term efficacy of PC and compare it with other treatment options in well-defined cohorts.

## 5. Conclusions

Our findings confirm that PC is a safe and effective treatment alternative for AC in high-risk patients. It has low complication and mortality rates. However, interval cholecystectomy, when possible, improves long-term outcomes. Further prospective studies are needed to define patient selection criteria, optimal drain removal time, and best timing of cholecystectomy after PC.

## Figures and Tables

**Table 1:** Basic characteristics and treatment outcomes of the 449 patients who underwent PC between January 2013 and May 2023.

Variable	All Patients, n = 449
Age, years, median (IQR)	80 (73.0–85.0)
Sex, n (%)	
Male	194 (43.2)
Female	255 (56.8)
ASA classification ^a^, n (%)	
ASA I	3 (0.7)
ASA II	44 (9.8)
ASA III	207 (46.1)
ASA IV	195 (43.4)
Sepsis, n (%)	
Presence of sepsis	104 (23.2)
TG18 severity of AC ^b^, n (%)	
Grade I	68 (15.1)
Grade II	280 (62.4)
Grade III	101 (22.5)
Type of cholecystitis, n (%)	
Calculous	424 (94.4)
Acalculous	25 (5.6)
Laboratory data on admission	
WBC ^c^ (×10^9^/L)	13.6 (10.5–18.0)
CRP ^d^ (mg/L)	161.6 (60.2–269.65)
Time to PC ^e^ insertion (median, IQR)	1 (1–2)
Admission day	59 (13.1)
Day 1	172 (38.3)
Day 2	77 (17.1)
Day 3	33 (7.3)
Others	108 (24.1)
Procedure complication, n (%)	
Dislocation	8 (1.8)
Occlusion	2 (0.4)
Bile leak	13 (2.9)
Bleeding	2 (0.4)
Other	12 (2.7)
Duration of PC treatment, days, median (IQR)	9 (6–14)
Clinical outcomes	
ICU ^f^ admission, n (%)	79 (17.6)
Hospital stay, days, median (IQR)	12 (9–15)
Readmission cause, n (%)	66 (14.7)
Pain	6 (1.3)
Recurrent AC	34 (7.6)
Acute pancreatitis, choledocholithiasis	19 (4.2)
Abscess (biloma)	3 (0.7)
Catheter disfunction	1 (0.2)
Others	3 (0.7)
Hospital mortality, n (%)	24 (5.3)

^a^ ASA: American Society of Anaesthesiologists Physical Status Classification System. ^b^ AC: Acute cholecystitis. ^c^ WBC: White blood cell count. ^d^ CRP: C-reactive protein. ^e^ PC: Percutaneous cholecystostomy. ^f^ ICU: Intensive care unit.

**Table 2:** Surgical outcomes.

Variable	All Patients, n = 174
Duration from drainage to index cholecystectomy, days, median (IQR)	7 (4–12)
Cholecystectomy approach, n (%)	
LC ^a^	81 (46.6)
OC ^b^	84 (48.3)
Conversion surgery	9 (5.2)
Complications after cholecystectomy, n (%)	6 (3.5)
BDI ^c^	3 (1.7)
Blood vessel injury	1 (0.6)
Bile leak	1 (0.6)
Wound eventration	1 (0.6)

^a^ LC: Laparoscopic cholecystectomy. ^b^ OC: Open cholecystectomy. ^c^ BDI: Bile duct injury.

**Table 3:** Comparative analysis of patient characteristics and clinical course between the definitive PC and bridging PC groups.

Variable	Definitive PC n = 275	Bridging PC n = 174	*p*-Value
Age, years, median (IQR)	81 (76–87)	75 (68–81)	<0.001
Sex, n (%)			0.182
Male	112 (40.7)	82 (47.1)	
Female	163 (59.3)	92 (52.9)	
ASA ^a^ classification, n (%)			<0.001
ASA I	0	3 (1.7)	
ASA II	16 (5.8)	28 (16.1)	
ASA III	108 (39.3)	99 (56.9)	
ASA IV	151 (54.9)	44 (25.3)	
Sepsis, n (%)			
Presence of sepsis	64 (23.3)	40 (23.0)	0.945
TG18 severity of AC ^b^, n (%)			0.454
Grade I	37 (13.5)	31 (17.8)	
Grade II	175 (63.6)	105 (60.3)	
Grade III	63 (22.9)	38 (21.8)	
Laboratory data on admission			
WBC ^c^ (×10^9^/L)	13.9 (10.5–17.6)	13.0 (10.0–18.4)	0.278
CRP ^d^ (mg/L)	174.1 (82.4–278.0)	125.0 (47.0–243.0)	0.003
Duration of PC treatment, days, median (IQR)	12 (7–14)	7 (5–11)	<0.001
ICU ^e^ admission, n (%)	45 (16.4)	34 (19.5)	0.389
Hospital stay, days, median (IQR)	11 (8–14)	13 (10–19)	<0.001
Hospital mortality, n (%)	23 (8.4)	1 (0.6)	<0.001
Recurrent AC	18 (6.5)	16 (9.2)	0.301

^a^ ASA: American Society of Anaesthesiologists Physical Status Classification System. ^b^ AC: Acute cholecystitis. ^c^ WBC: White blood cell count. ^d^ CRP: C-reactive protein. ^e^ ICU: Intensive care unit.

**Table 4:** Univariate and multivariate analysis for risk factors related to in-hospital mortality.

Characteristics	Univariate		Multivariate	
	HR (95% CI)	*p*-Value	HR (95% CI)	*p*-Value
Sex				
Male	reference			
Female	0.64 (0.29–1.44)	0.282		
ASA ^a^ classification				
ASA I	reference			
ASA II	1.0 (0.0–5.37)	>0.99		
ASA III	442.26 (0.0–3.15)	0.952		
ASA IV	3286.33 (0.0–2.33)	0.936		
Sepsis				
Presence of sepsis	8.06 (3.34–19.43)	<0.001	9.64 (1.31–70.77)	0.026
TG18 severity				
Grade I	reference		reference	
Grade II	1.7 (0.21–13.82)	0.620	1.96 (0.23–16.8)	0.539
Grade III	10.77 (1.43–81.23)	0.021	1.49 (0.14–16.48)	0.745
Treatment strategy				
PC ^b^ alone	reference		reference	
PC with cholecystectomy	0.07 (0.01–0.51)	0.009	0.07 (0.01–0.51)	0.009

^a^ ASA: American Society of Anaesthesiologists Physical Status Classification System. ^b^ PC: Percutaneous cholecystostomy.

## Data Availability

The underlying dataset is available for non-commercial purposes from the corresponding author upon reasonable request.
